# Palliative home parenteral nutrition in patients with ovarian cancer and malignant bowel obstruction: experiences of women and family caregivers

**DOI:** 10.1186/s12904-019-0507-5

**Published:** 2019-12-29

**Authors:** Anne Marie Sowerbutts, Simon Lal, Jana Sremanakova, Andrew R. Clamp, Gordon C. Jayson, Antje Teubner, Lisa Hardy, Chris Todd, Anne-Marie Raftery, Eileen Sutton, Robert D. Morgan, Alexander J. Vickers, Sorrel Burden

**Affiliations:** 1 Faculty of Biology, Medicine and Health and Manchester Academic Health Science Centre, University of Manchester, Manchester, UK; 2 School of Health Sciences, University of Manchester, RM5.328 Jean McFarlane Building, Oxford Rd, Manchester, M13 9PL UK; 3 Salford Royal NHS Foundation Trust, Manchester, UK; 4 The Christie NHS Foundation Trust, Manchester, UK; 5 Manchester University NHS Foundation Trust, Manchester, UK; 6 Department of Social Medicine, University of Bristol, Bristol, UK

**Keywords:** Parenteral nutrition, Ovarian cancer, Bowel obstruction, Phenomenology, Qualitative

## Abstract

**Background:**

Malnutrition is a problem in advanced cancer, particularly ovarian cancer where malignant bowel obstruction (MBO) is a frequent complication. Parenteral nutrition is the only way these patients can received adequate nutrition and is a principal indication for palliative home parenteral nutrition (HPN). Giving HPN is contentious as it may increase the burden on patients. This study investigates patients’ and family caregivers’ experiences of HPN, alongside nutritional status and survival in patients with ovarian cancer and MBO.

**Methods:**

This mixed methods study collected data on participant characteristics, clinical details and body composition using computed tomography (CT) combined with longitudinal in-depth interviews underpinned by phenomenological principles. The cohort comprised 38 women with ovarian cancer and inoperable MBO admitted (10/2016 to 12/ 2017) to a tertiary referral hospital. Longitudinal interviews (*n* = 57) were carried out with 20 women considered for HPN and 13 of their family caregivers.

**Results:**

Of the 38 women, 32 received parenteral nutrition (PN) in hospital and 17 were discharged on HPN. Nutritional status was poor with 31 of 33 women who had a CT scan having low muscle mass, although 10 were obese. Median overall survival from admission with MBO for all 38 women was 70 days (range 8–506) and for those 17 on HPN was 156 days (range 46–506).

Women experienced HPN as one facet of their illness, but viewed it as a “lifeline” that allowed them to live outside hospital. Nevertheless, HPN treatment came with losses including erosion of normality through an impact on activities of daily living and dealing with the bureaucracy surrounding the process. Family caregivers coped but were often left in an emotionally vulnerable state.

**Conclusions:**

Women and family caregivers reported that the inconvenience and disruption caused by HPN was worth the extended time they had at home.

## Introduction

Malnutrition is a problem in advanced cancer, affecting up to 80% of patients [1]. It is a particular problem in advanced ovarian cancer as a complication affecting between 20 and 50% of patients is malignant bowel obstruction (MBO) [2, 3]. In MBO, the gastrointestinal system is unable to function normally and the patient’s ability to digest food and fluids is minimal. The only way for these patients to receive adequate nutrition is via parenteral nutrition (PN).

The numbers of patients with cancer receiving home parenteral nutrition (HPN) in the UK is increasing, particularly in patients with ovarian cancer [4]. Given these increasing numbers it is important to know the objective and subjective benefits. Studies have pointed to improvements of nutritional status for these patients [5]; however, clear benefits in terms of survival and quality of life for patients with MBO have yet to be definitively demonstrated. A systematic review examining survival and quality of life of HPN for people with inoperable MBO found the quality of evidence, as assessed using GRADE [6], very low and therefore the effect of HPN on survival and quality of life continues to be inconclusive [7].

As clear benefits remain equivocal and these patients have palliative care needs, it is particularly important to know patient and family carers subjective experience. To date studies examining patient and family experience have not directly addressed the situation of people in MBO receiving HPN, who are unable to administer HPN themselves due to their state of ill health and it is the sole source of nutrition given daily [8, 9].

The aim of this study was to investigate the experience of HPN for women with ovarian cancer and MBO and their family members acting as caregivers, in the context of the nutritional status and survival of a cohort of patients with ovarian cancer and MBO.

## Materials and methods

### Study design and participant recruitment

This was a mixed method study which included data on participant characteristics, body composition and clinical details alongside longitudinal in-depth interviews underpinned by phenomenological principles [10]. The study cohort was comprised of 38 women admitted with ovarian cancer and inoperable MBO to a tertiary referral cancer hospital in the North of England between October 2016 and December 2017. In total 20 women and 13 family caregivers took part in 57 interviews. Patients were recruited from the tertiary referral centre and family caregivers were recruited by asking patients to nominate a relative. Informed consent was collected from participants. The study was approved by East of England Cambridge Central Research Ethics Committee on behalf of the Health Research Authority National Research Ethics Service.

### Study inclusion criteria

**Fig. 1:**
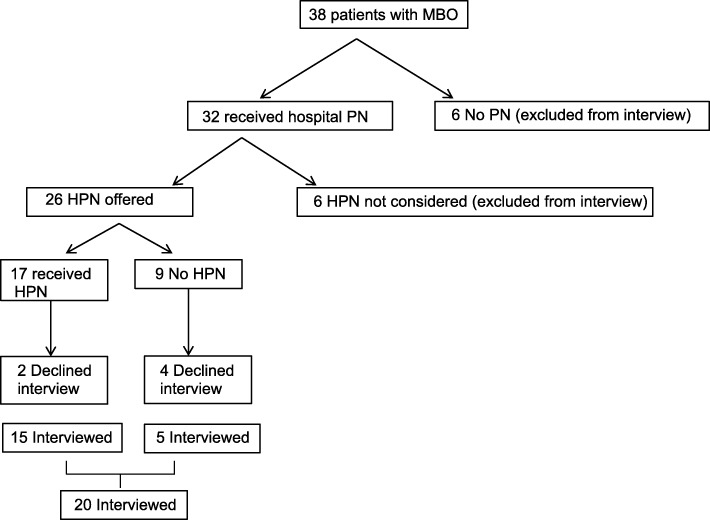
Flow chart of patients with MBO considered for HPN taking part in an interview. PN = Parenteral Nutrition. HPN = Home Parenteral Nutrition

Women with ovarian cancer and inoperable MBO receiving palliative chemotherapy or best supportive care were included. Patients were considered to have MBO after review of appropriate cross-sectional imaging by an oncological radiologist with extensive experience in the management of ovarian cancer. Findings considered inoperable included multilevel obstruction, extensive serosal disease with no transition point, extensive mesenteric disease which would prevent bowel mobilisation. A gastrointestinal surgical consultation took place in cases where there was uncertainty regarding operability. Those who were considered eligible to receive HPN (26 patients – see Fig. 1) were invited to take part in interviews; ability to provide informed consent and converse in English were required.

### Context

Patients were started on PN on a ward at a specialised oncology hospital. HPN discharge was overseen remotely by a specialist intestinal failure unit situated at another hospital across the city. The two centres aimed to discharge medically fit patients within 2 weeks. Home discharge took place when, patients’ symptoms related to MBO were adequately controlled, there was satisfactory fluid and electrolyte balance with an established PN prescription, the patient’s performance status was stable and any recommended home care package was in place. A homecare company supplied equipment, feed, and employed nurses to connect and disconnect feed. Once home, patients had a two-hour slot each evening and morning for nurses to administer and discontinue infusions. The PN was infused overnight, every night. No other medication was given by the PN central venous catheter.

### Data collection

#### Quantitative data

Data were collected on patients’ characteristics at the time of MBO and outcomes that included survival and total length of time on PN and HPN. Nutritional status was measured using self-reported height and usual body weight, weight at the start of PN (Seca scales) or, if unavailable, estimated dry weight and body mass index.

Body composition was assessed by analysing the computed tomography (CT) image taken nearest to the time of admission with MBO using software (SliceOMatic, Tomovision, Canada). Single axial images at the level of the third lumbar (L3) vertebrae were used to measure total skeletal muscle and total fat mass at the cross-sectional area. Hounsfield unit thresholds were set at − 190 to − 30 for subcutaneous fat, − 150 to − 50 for visceral fat, − 190 to − 30 for intramuscular fat and − 29 to 150 for skeletal muscle. The muscle mass and fat mass were calculated using a standard equation [11]. A low muscle mass was defined as < 38.9cm^2^/m^2^ at the L3 muscle [12]. Obesity was defined as a visceral fat mass above 80.1 cm^2^ [13].

#### Qualitative data

Women were interviewed up to four time points; in hospital and subsequently up to three times whilst receiving HPN. Family caregivers were also interviewed a maximum of four times. Different topic guides were used with patients and family caregivers; topic guides were used flexibly allowing the interviewer to explore themes raised by the interviewee. With participant permission, interviews were audio-recorded and encrypted at source; interviews lasted up to 1 h. Interviews were transcribed verbatim.

Data collection continued until saturation of themes was achieved; determined by analysing data concurrently in an iterative process of reading and analysing transcripts. Data collection analysis was underpinned by hermeneutic phenomenology allowing a transition from the descriptive to interpretive paradigm [10]. The phenomenon under investigation was the lived experiences of women on HPN and the experiences of family caregivers.

### Data analysis

#### Statistical analysis

Descriptive statistics were used to describe participants’ data using SPSS Version 22 (IBM Corp., Armonk, NY, United States) software.

#### Qualitative data

Interview transcripts were managed using NVivo Version 11 (QSR International Pty Ltd., Doncaster, VIC, Australia) and they were analysed thematically influenced by principles described by Van Manen [14]. Transcripts were iteratively read to identify and code themes found in the text. Reading progressed from interpreting the whole interview to selecting phrases (thematic of the experience of HPN), to a detailed line by line coding. From these codes explanatory themes were created. Similar themes were grouped together to form a hierarchy of themes and subthemes. Anonymised quotations were taken from the text for each theme and used in this paper to show a flavour of the data. Reflection on the meaning of the text in context was undertaken to capture motivations, emotions, and drivers from participants to add an interpretive dimension to the analytical process.

Rigour was introduced by using reflexivity with field notes; two researchers (AMS and ES) developed the coding framework and double coded 10% of interviews. The longitudinal nature of the interviews allowed an opportunity for participants to reflect on their previous interviews, facilitating clarification of meaning and documentation of changes in their attitudes over time. Moreover, the interpretation of the data was checked with three women and five family caregivers to ensure the analysis reflected their experience.

## Results

Over a 15-month period, 38 women with MBO were identified for inclusion from the clinical recruitment site in this study, patient characteristics are shown in Table 1. The median interval from initial diagnosis of ovarian cancer to the development of bowel obstruction was 27.5 months (range 1–184 months). More than half (22 patients) were considered to have a good performance status, performance status 0 to 2, whereas 17 patients had unknown or poor performance status; performance status 3 and 4. From these 38 women, 26 women were considered for HPN and 20 were interviewed (Fig. 1). None of the patients who received HPN developed a line infection. Characteristics were similar between those women interviewed and those who were not interviewed. In addition, 13 family caregivers were recruited. A total of 57 interviews were conducted, with participants being interviewed up to four times.

**Table 1:** Patient characteristics at the time of bowel obstruction

	Interviewed (*n* = 20)	Not interviewed (*n* = 18)	*N* (%)
Age, mean ± SD	67 (7.5)	64 (10.1)	
Stage at diagnosis			
Stage 1	1	2	3 (7.9)
Stage 2	0	1	1 (2.6)
Stage 3	16	15	31 (81.6)
Stage 4	3	0	3 (7.9)
Missing	0	0	0
Histology at diagnosis			
Serous	15	16	31 (81.6)
Endometrioid	1	0	1 (2.6)
Clear cell	1	1	2 (5.3)
Mucinous	0	1	1 (2.6)
Adenocarcinoma NOS	2	0	2 (5.3)
Carcinosarcoma	1	0	1 (2.6)
Previous surgery			
Total anterior hysterectomy	18	14	32 (82.5)
Bilateral-salpingo-oophorectomy	17	12	29 (76.3)
Omentectomy	15	14	29 (76.3)
Re-laparotomy	4	4	8 (20.5)
Chemotherapy at BO admission			
Total number	12	13	25 (63.0)
Platinum based	7	7	14 (37.0)
ECOG PS at BO admission			
0	1	0	1 (2.6)
1	5	3	8 (21.1)
2	7	5	12 (31.6)
3	3	4	7 (18.4)
4	0	1	1 (2.6)
Unknown	4	5	9 (23.7)

No statistical significant differences

*ECOG PS* Eastern Cooperative Oncology Group performance status

### Quantitative data

#### Nutritional status

Nutritional status of the women at the time of MBO was poor; body composition measured on CT scans showed that 30/33 had low muscle mass, and 10/33 women were obese. CT scans were unavailable for five women. The mean time between CT scan and date of discharge was 53.41 days (SD ± 63.9). The Glasgow prognostic score, a marker of systematic inflammation used to predict survival in cancer with a higher score associated with poorer prognosis, was 2 in 21 women, 1 in 14 and 0 in 3 [15]. Details regarding nutritional status are presented in Table 2.

**Table 2:** Nutritional status of patients with ovarian cancer at the time of bowel obstruction

	Interviewed (*n* = 18) Mean ± SD	Not interviewed (*n* = 15) Mean ± SD	Total (*n* = 33) Mean ± SD
Fat mass (kg)	21.07 (5.86)	22.94 (7.55)	22.10 (6.7)
Muscle mass (kg)	30.79 (2.88)	32.77 (5.18)	31.83 (4.2)
Muscle Index (cm^2^/m^2^)	31.51 (4.49)	33.54 (6.01)	32.59 (5.3)
Missing CT scans	2	3	5
Body Mass Index (kg/m^2^)	24.10 (6.41)	23.06 (3.72)	23.67 (5.42)
BMI missing	0	4	4
C-reactive protein (mg/l)	62.84 (93.59)	73.44 (67.91)	67.86 (81.5)
Albumin (g/l)	33.10 (6.14)	36.89 (6.40)	34.89 (6.5)
Missing blood results	0	0	0

#### Parenteral nutrition and survival

Of the 38 women in the cohort, 32 received PN; infusions were daily and self-reported oral intake was minimal. From 32 patients who received PN, 4 died before discharge, 17 were discharged on HPN and 11 left without HPN. For the patients that were discharged without HPN, the reasons were: end of life care (*n* = 4), assessed as unsuitable by medical team (*n* = 3) and patient offered HPN but declined (*n* = 4). Median length of receipt of PN for all 32 women was 54 days (range 2 to 501) and median length on HPN for 17 women was 91 days (range 6 to 441).

**Fig. 2:**
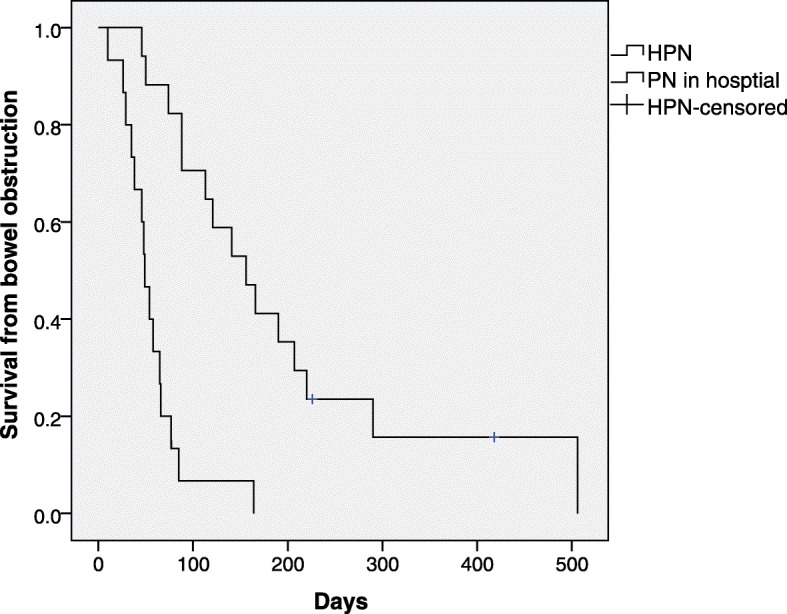
Survival of patients receiving parenteral nutrition in hospital compared to receiving parenteral nutrition at hospital and home. HPN = Home Parenteral Nutrition. PN = Parenteral Nutrition

At the study conclusion 36 of 38 women had died. Median survival from admission with MBO for all 38 women was 70 days (range 8 to 506). Survival from admission with MBO for all the 32 women who received PN was 81 days (range 10 to 506), for the 17 patients who had HPN was 156 days (range 46–506) (see Fig. 2) and for 6 women who did not receive PN was 20 days (range 8 to 109).

### Interviews

In total 20 women were interviewed; 11 in hospital and 9 in hospital and up to three times at home (Table 3). Family caregivers caring for the patients were interviewed on their own where possible so they could freely express their views; 17 interviews with 13 family caregivers were conducted without the woman present.

**Table 3:** Time, place and numbers of patient and caregivers taking part at each time point

	Interview 1	Interview 2	Interview 3	Interview 4	Total
Time from initial discharge		30–48 days	70–110 days	251–329 days	
Patient in Hospital (n)	20			1	21
Patient at Home (n)		9	7	2	18
Total					39
Carer at hospital (n)	8				8
Carer at home (n)	5	8	3	1	17
Total					25

*n* = number of patients or carers taking part at each time point. Thirteen carers and 20 patients took part in 57 interviews. Seven interviews involved more than one person; either family caregivers were interviewed together or family caregivers and patients

### Themes

Four major themes were identified from the data; “*competing priorities*” as patients experienced PN as one facet of their illness, “*gains*”, “*losses of normality*” and “*balancing losses and gains*.” Quotations representative of the emergent themes are given.. Pseudonyms are used for all participants. For the family caregivers their relationship to the women is also indicated. Further exemplars of the themes are given in Additional file 1, all names used are pseudonyms.

#### Theme 1 competing priorities

Women described the complexity of their ovarian cancer journey. They had had many treatments, appointments, major setbacks and minor annoyances. In one sense HPN was just another facet of their illness and additional experience competing for attention amongst many. For example, one woman described the irritation she had with her GP’s surgery. “*I sent a sample off to the doctor on Thursday and they just sat on it for four days … so I’m a bit annoyed. That’s nothing to do with the feed*” (Caroline).

When PN was started in hospital it was unexceptional for most women, as they generally expressed the view that in this environment it was normal to undergo interventions and to be surrounded by medical equipment. On the ward, PN was administered by the nurse as are other fluids or drugs and in this setting it is unremarkable: “*they come along and they flush it, and then they set something up on the drip and they will say, that is ready for going on*” (Betty). One exception was a patient who focused her anger about not eating on the PN as she felt receiving nutrition in this way was similar to being fed like a horse with a nosebag.

In contrast to their experience in hospital, on discharge HPN did stand out as it was unusual to have medical procedures at home: “*initially when this was being discussed with us … I thought it was probably less medical than what it is*” (Alice, daughter).

#### Theme 2 gains

##### Survival

Both women and family caregivers viewed HPN as offering gains. For women, the main advantage was the extra life it gave them, and this remained so throughout the course of the interviews: “*If I don’t have the [HPN], I won’t be here. There’s no other way, is there?*” (Kirsten). Women viewed HPN as a ‘lifeline.’ If they wanted to continue living then nutrition was necessary and HPN was the only way that patients could receive it: “*It’s either die with food or [HPN] for the rest of your days and I’d sooner live and be on [HPN]*” (Sam).

##### Quality of life

Women perceived HPN as not only improving their quantity, but also quality of life as they could be out of hospital and at home with friends and family. As one patient commented: “*spending time with family when you get to, like, my stage, is the most important for everybody*” (Sylvia).

Being at home meant that patients were able to achieve an element of normality in their lives. It was important for patients having aspects of life that were unchanged by their illness. Patients were keen to maintain their ordinary routine and valued any activities of daily living they were able to manage, for example one patient appreciated being able to get out into the garden “*I went in the garden the other day and did a bit of tidying up*” (Laura).

Receiving HPN enabled patients to engage in enjoyable activities, sometimes outside of the home. For a subset of patients, these activities were goals that they wanted to achieve such as preparing for Christmas or organising a family party: “*I’ve sorted all Christmas out*” (Stacey).

Some patients discussed HPN as having health benefits. They commented that it gave them more energy or strength: “*it’s given me, yes, more energy*” (Mabel). Some thought it stabilised or increased their weight: “*I think I’ve put a little bit of weight on*” (Penny).

### Family caregivers

Family caregivers echoed the views of patients in that they saw the principal gain as allowing their relative extra time. As one husband said “*It’s keeping her alive really. That’s the big advantage*.” (John, husband). Two husbands saw it as giving their wives strength or energy: “*it’s going to help her do what she wants to do*” (Derek, husband).

Caregivers were also appreciative that the treatment could be given at home as this would facilitate normality: “*I’m looking forward to her being able to come out of hospital and go home and have the [HPN] at home and, sort of, have some sort of normality to life*” (Alice, daughter); for example making it easier for friends and neighbours to visit: “*our friends and neighbours and relatives can get easy access to her*” (Joseph, husband).

For the family caregivers themselves, the patient being at home meant they were saved from travelling into the hospital. For three family caregivers this was a big advantage as they either lived a long distance away or could not travel there easily on public transport.

#### Theme 3 loss of normality

However, the gains that patients and family caregivers perceived from HPN came at the expense of many losses to normality.

##### Curtailment of activities of daily living

Disease progression and HPN gradually eroded patients’ ability to undertake activities of daily living. A massive loss in patients’ lives was eating as having MBO meant their oral intake was minimal.

Disruption to sleep was another problem faced by patients. HPN was given overnight and patients received up to three litres of fluid. This volume meant that some patients needed to make frequent trips to the toilet “*It would be wonderful if I could have even 5 h sleep without a break*” (Marilyn).

Although some patients managed to go out, on the whole patients’ social lives were curtailed. Some only left the house for hospital appointments as they did not feel well enough to go anywhere else. Others would occasionally go out, but for as short a time as possible as they had a nasogastric tube in place and found it challenging when people stared at them.

##### Limiting bodily freedom

Patients’ freedom of movement and control of their own bodies were limited by the physical attachment to HPN and drainage bags. When commencing HPN, the weight of the feed was a big issue: “*It’s difficult, yeah, especially going upstairs, because I’ve not got much energy, so usually my husband … has to take it for me*” (Caroline). Even in the morning when the bag was empty patients found being attached to it restricting. One family caregiver referred to it as “*a ball and chain*” (John, husband).

Moving about attached to tubes and bags was most problematic for frailer patients. These patients commented that health care professionals would underplay the problems that they would have at home: “*It wasn’t as easy as it was made out to be*” (Belinda).

However, some patients found the most restrictive aspect was their venting gastrostomy. The HPN was only attached overnight whereas they were only free from the gastrostomy when they were showering: “*when I go in the shower and everything, I can … take both tubes off, and I’m a different person*” (Kirsten).

##### Imposed routine

In addition to losing their own routine, patients were subjected to new routines not of their choosing. They had to deal with the bureaucracy surrounding healthcare services. The patient or family caregiver needed to be available to receive deliveries of feed. Patients also had healthcare appointments to attend, with their oncologist and for monitoring their HPN.

However, the main restriction was the nurses’ home visits and the patients’ days were scheduled around those morning and evening visits. Patients found the nurses cheerful and efficient and no one was critical of nursing care. Nevertheless, for some the mere presence of the nurse was a twice daily reminder that they were ill and the clinical environment imposing itself on their home.

Despite the loss of the patients’ own routines, new routines were established and became accepted. “*It just becomes a way of life really, you know what I mean, this is how your day goes and this is what it is. A nurse comes and takes it off in a morning and then a nurse comes at night and puts it back on*” (Belinda).

##### Changes to the meaning of home

HPN introduced a myriad of equipment into the home so that it became a medical environment. One patient was shocked at the amount of equipment even though she had been told about it. Prior to starting HPN, some women were concerned where the equipment would go, although they subsequently managed to accommodate it.

##### Changing relationships

Some women’s relationships changed when their relative became their main carer. For most of the women this was their husband. As the women became unwell they were unable to carry out some household tasks such as cooking. These tasks fell to the family caregiver alongside physically caring for the patient, although assistance was provided by district nurses and healthcare assistants for frailer patients. Several patients commented that their relative did not mind doing this for them “*I’ve done things for him. He can do things for me*” (Michelle), although some recognised that it caused a strain on their family member. One patient was keen to protect her husband as much as possible, remarking about her night-time bathroom visits “*I’ve tried creeping, ‘cause I don’t want to wake him up*” (Penny). Her husband on the other hand was keen to help: “*I’m awake most of the night listening for her, but she tells me not to help her*” (Simon, husband).

### Family caregivers

Overall, family caregivers thought HPN was beneficial for their relative. However, HPN meant that they had to contend with various losses. Family caregivers reported a physical toll, for example if they were frail themselves, carrying heavy HPN bags for patients could be problematic. Some family caregivers reported financial implications, particularly if they were self-employed: “*as for going out and taking a contract on or something. It’s just not feasible*” (John, husband). Free time for family caregivers was limited. They were restricted from going out by the number of healthcare professionals coming to their home. If the patient and family caregiver did manage to get out they needed to be back in time for the HPN nurses’ arrival. In addition, there were social impacts. For example, as the patient could not eat, husbands ate alone and some had to learn to cook for the first time: “*Not my strong point … No, I burn water*” (Chris, husband). Supporting their relative led to some family caregivers neglecting their own health: “*I am physically falling to bits*” (Scarlett, daughter).

Family caregivers wanted to care for their relative, but this could sometimes be difficult leading to contradictory statements or attitudes. One husband said that caring is “*what you sign on for when you get married*” (Simon, husband). However, at the end of the second interview, he reported feeling like a “*prisoner*” being very limited in the time that he could go out. Moreover, there were two families who said that they wanted their relative home as soon as possible. However, when the discharge process was started, they brought up questions related to the fitness of their relative to cope at home.

Some family caregivers wanted more information. They felt they received information in an ad hoc manner if they were present when a healthcare professional visited a patient, but otherwise they could miss out: “*facts and information had been given to my mum that I hadn’t been privy [party to] to, I think I should have been privy [party to] to it*” (Alice, daughter).

#### Theme 4 balancing gains and losses

##### Stopping HPN

Given the losses experienced, patients were asked if they had thought about stopping the HPN. Stopping HPN was largely mentioned in a general way by oncologists, but patients did not seem to take this on board as they were surprised by the question. Seven of the nine patients having HPN were unequivocal during all the interviews they would not stop it: “*No, why would I do that?*” (Laura). The reasons cited by four were they would be dead or it is keeping them alive. One said that they would be hungry and another that it had become a way of life. One patient would perhaps think about it if she became very unwell in the future: referring to that time she said: “*when I get to the point where I’ve got to say, oh, enough is enough, it won’t matter then, but until that point comes then I just have to fight, keep going*” (Stacey). This patient was unusual as a doctor at the intestinal failure unit had asked her to think about what she wanted to do in the event of her developing sepsis from a central venous catheter infection. Another patient, during her second interview, referring to stopping, said “*I do get the odd moment when I feel really tired*” (Marilyn). However, it was not something that she had seriously considered. In subsequent interviews when she was feeling a bit better, she did not entertain the idea of stopping.

##### Hope

The theme of hope was useful for examining how patients viewed their lives along their journey of HPN. Early in the process, some patients were still receiving chemotherapy and hoped that their condition would improve sufficiently be able to eat again. Such patients viewed this as a genuine possibility.

Later on, patients hoped for things in a more wistful way. For example, one patient said she would like to go on holiday, but realised that this was not possible at her stage of illness. Another said that she hoped some treatment might be found to deal with the MBO.

Other patients at this stage had more contained or realistic hopes. One patient hoped for a bit more time and another that she would be pain free at the end. In contrast, one woman said that she did not hope for anything. However, on further discussion she was planning a night away with her two daughters indicating she was looking forward.

## Discussion

This is the first time, to our knowledge, that longitudinal in-depth interviews have been carried out with people in MBO receiving HPN, and placed in the context of nutritional status and survival of a cohort of similar ovarian cancer patients. Patients and family caregivers in this study viewed HPN as offering benefits in terms of survival and improving quality of life by enabling patients to be out of the hospital environment. However, these benefits were accompanied by many losses to normality. Overall the experience of HPN for patients and family caregivers was bittersweet bringing both advantages and restrictions.

Patients viewed HPN as the lifeline enabling them to continue living. They had a median of around 3 months on HPN, which concurs with data from other studies which found a median overall survival of between 60 to 155 days (range 2 to 1278 days) [16–19]. Historically, it has been found that people are unable to live longer than approximately 70 days without nutrition [20–23]. Therefore, it is possible that patients living longer than this period were correctly assuming HPN added to their lifespan as oral intake was minimal and nutritional needs were met primarily through HPN.

Interestingly, patients achieved on average 3 months at home despite the majority having poor nutritional status, which is known to reduce survival [24]. This length of survival is in line with current guidance for considering HPN in advanced cancer patients [25], which also recommends considering patient quality of life and using performance measures as an indicator for HPN. In this cohort over half of the patients had a relatively good performance status at the time of MBO diagnosis.

For the patients who got home, HPN improved individuals’ quality of life by enabling them to be out of hospital. This allowed patients to have some normality in their lives which was appreciated possibly because their illness had eroded so much of their previous way of life. These experiences echo those of patients with benign intestinal failure on HPN who discuss HPN as a lifeline, which allows them some normality [8, 26]. There are also similarities between our findings and patients with advanced cancer not in MBO having HPN as supplementary nutrition. As with some of the women in our cohort, HPN was reported as increasing energy and strength while preventing weight loss [9, 27].

Whilst HPN offered gains, patients also experienced many losses of normality. Women viewed these losses in the context of their illness as a whole, but it was clear that the losses may have been disease-related such as loss of eating or HPN-specific such as being connected to the infusion. The theme of loss and the particular losses women faced resonated with those discussed in the literature. HPN is experienced in the context of living with other symptoms of illness, which may be burdensome [8, 26, 28]. Other recurring themes are restrictions imposed from being connected to the HPN and the lack of autonomous control over their body [8, 26, 29]. Moving about with the feed was particularly problematic for frailer patients. For these patients, healthcare professionals need to be realistic rather than too optimistic about the difficulties ahead when discussing HPN.

In order to assess how burdensome HPN was for patients, they were asked if they had thought about stopping it, as patients may consider stopping a treatment that they find troublesome or that is associated with poor quality of life [30]. However, for these patients the burden of treatment did not outweigh benefit. Two patients who reflected on stopping did not seriously consider it as an option at that point. Most immediately dismissed the idea, which is consistent with other studies, which have demonstrated that patients with advanced cancer do not find HPN more burdensome than those with benign disease [31].

Hope is an important element for cancer patients coping with their illness [32]. Studies have found hope to be high even in advanced cancer patients and loss of hope being associated with a decreased ability to cope [33, 34]. The patients in this study had hopes, although overtime this changed from being unrealistic to more circumspect. However, engaging in hope indicated that patients were actively living their lives. This is consistent with other work, which reported that terminally ill patients thought of themselves as living while dying as long as they could still participate in day to day activities [35].

Family caregivers agreed with the patients’ views of the benefits of HPN. However, the burden of caring led some family caregivers to express feelings of ambivalence. Carer burden is widely acknowledged and it is recognised that for some this can give rise to mixed emotions [36, 37]. Also prominent in the literature is the need to involve family caregivers in clinical decision making [38]. Currently, family caregivers only receive information if they happen to be present at a bed-side consultation. Given the impact of HPN on family caregivers it may be preferable that they have the opportunity to be present at discussions relating to the practicalities of treatment.

A strength of this work is the use of longitudinal interviews. This method allowed the capture of patient and family caregiver experiences and changes in attitudes over time. Given that all the patients had palliative care needs the recruitment rate was very high. Moreover, we collected quantitative data on patient characteristics, body composition and survival to place the interviews in context. However, a limitation of the study was that the data collected was from patient diagnosed with bowel obstruction at one tertiary referral centre and further work would be needed to show whether it could be generalisable. Other limitations that this caused were that as surgery was not performed at the oncology hospital it was only possible to collect data on the type of operation. It was not possible to collect accurate data about whether procedures were performed at the same time or on different occasions. Also, information has been given on infection rates as a complication patients may encounter with HPN; however, data has not been included on all hospital readmissions as patients may have been admitted to a hospital local to their home for cancer related issues.

## Conclusion

In conclusion, from patients’ experiences burden of treatment did not mitigate the benefits of HPN. Motivation to live outweighed the constraints imposed. Patients and family caregivers recognise the treatment as a lifeline and are grateful for it. However, healthcare professionals need to be alert to the losses that patients will face and present a realistic picture of them. In the provision of HPN family caregiver needs and ability to cope over time also need to be considered. Nevertheless, patients were prepared to suffer losses to continue living.

## Supplementary information

**Additional file 1.** Quotations from women with ovarian cancer receiving parenteral nutrition and their family caregivers. Additional quotes from patients and family members to support the themes given in the results section.

## Data Availability

The datasets used and analysed during the current study are available from the corresponding author on reasonable request.

## Abbreviations

CT: Computed tomography
HPN: Home parenteral nutrition
MBO: Malignant bowel obstruction
PN: Parenteral nutrition
