# Supplementary material for: Palliative home parenteral nutrition in patients with ovarian cancer and malignant bowel obstruction: experiences of women and family caregivers
Source: BMC Palliat Care. 2019 Dec 29;18:120. doi: 10.1186/s12904-019-0507-5 (PMC6936090; doi:10.1186/s12904-019-0507-5)
Supplement: Supplementary file 1 — Additional file 1. Quotations from women with ovarian cancer receiving parenteral nutrition and their family caregivers. Additional quotes from patients and family members to support the themes given in the results section. [file 12904_2019_507_MOESM1_ESM.docx]

**Supplementary material 1: Quotations from women with ovarian cancer receiving parenteral nutrition and their family caregivers**

| **Theme: Subtheme** |
| --- |
| **Theme 1 Competing priorities** |
| “[it’s] just something else dripping into my system isn’t it? Here it’s just one more thing going through” (Michelle) . |
| “in the whole scheme of things for me, there’s lots going on; it’s not been a massive big deal really.” (Hilary) |
| I have been a bit (*downhearted*), but it’s……it’s not through that (*HPN)*, I think it’s just …because I’ve not felt well…you’ve got this (*nasogastric tube*), and it’ll get hooked on everything…at night when you’re getting up for the loo, you’ve got that in one hand (*drainage bag)* and then you’ve got that (*HPN bag)* in the other...I’m not sleeping proper anyway, but I’ve had a few things, like I’ve got the results of my scan tomorrow so I’ve got that going round in my mind (Kirsten) |
| **Theme 2 Gains: Survival** |
| “if I hadn’t have had it I would be dead by now “ (Laura) |
| “Well, I mean, I don’t think I'd have survived eating anything…I was so sick when I first went into hospital, literally, vomiting quite a lot of the time. I wouldn’t have been able to take any feed other than something like that, so it's been a massive advantage, massive.” (Hilary) |
| **Theme 2 Gains: Quality of life** |
| “Obviously having it in your own home is even more of a plus point, isn't it?” (Paul, husband) |
| “I have, yeah. I get dressed and when you're in the hospital you're in a nightdress all day and it's just nice to get dressed…Yeah, it means a lot getting dressed, it's normality, isn't it? I like normality, yeah” (Penny) |
| “Well, I have written letters. I’ve roughly written letters for my sons and my dad, so I want, like, to write those up properly. So, that has given me time that I can do that with as well.” (Sylvia) |
| **Theme 3 Losses: Curtailment of activities of daily living** |
| “I end up getting up in the middle of the night because there’s a lot of liquid to take in so normally I wake about three and then I have to go through to the [toilet]” (Stacey) |
| **Theme 3 Losses: Limiting bodily freedom** |
| I stay downstairs until Scarlett (daughter) comes and then she brings it up and we just put it here at the side of the bed (Belinda) |
| **Theme 3 Losses: Imposed routines** |
| it gets put on and then took off in morning; there’s nothing any different in that respect (Kirsten). |
| “I think the biggest problem is the timing…They come too early in the afternoon“ (Caroline) |
| **Theme 3 Losses: Changes in meaning of home** |
| It was a bit of a shock when you see all the fridge and everything and all the equipment, but they did warn me. (Caroline) |
| **Theme 3 Losses: Changing relationships** |
| But he’s been a diamond. He has been a husband in a million…I never thought that I would get that out of my husband…I’m his patient. Plus his wife (Sam) |
| **Theme 3 Losses: Family caregiver – Financial** |
| Financially it’s not great. And you just want to keep on top of things, you know. It’s quite tough (John, husband) |
| **Theme 3 Losses: Family caregiver – Time** |
| It is a full day, there’s always something (John, husband) |
| **Theme 3 Losses: Family caregiver – Cooking** |
| “I've learnt how to cook…well it's the beans on toast a la carte or I can do fish and steamed vegetables, that's about it” (Phil, husband) |
| **Theme 4 Balancing gains and losses: Stopping parenteral nutrition** |
| Oh I don’t stop it, no. If I stop that I’m dead. It is my lifeline isn’t it? (Sam) |
| **Theme 4 Balancing gains and losses: Hope** |
| “I want to go back to Egypt, but that’s not going to happen is it?” (Sam). |
| “I hope that they might find a way of doing something with this obstruction I’ve got. In the future they might be able to do something about it.” (Laura). |
| “Just to be pain free” (Penny) |
